# Aqueous and ethanolic extracts of welsh onion, *Allium fistulosum*, attenuate high-fat diet-induced obesity

**DOI:** 10.1186/s12906-018-2152-6

**Published:** 2018-03-20

**Authors:** Yoon-Young Sung, Dong-Seon Kim, Seung-Hyung Kim, Ho Kyoung Kim

**Affiliations:** 1 Mibyeong Research Center, Korea Institute of Oriental Medicine, 1672 Yuseong-daero, Yuseong-gu, Daejeon, 305-811 Republic of Korea; 2 KM Convergence Research Division, Korea Institute of Oriental Medicine, Daejeon, 305-811 Republic of Korea; 3 Institute of Traditional Medicine and Bioscience, Daejeon University, Daejeon, 300-716 Republic of Korea

**Keywords:** Adiponectin, *Allium fistulosum*, AMP-activated protein kinase

## Abstract

**Background:**

*Allium fistulosum* (Welsh onion) is a traditional medicinal plant used for the treatment of colds, influenza, abdominal pain, headache, and heart disease. This study evaluated the effects of *A. fistulosum* ethanolic extract (AFE) and aqueous extract (AFW) on body weight and other obesity-related parameters.

**Methods:**

Male 8-week-old C57BL/6 J mice were fed either a standard chow diet (normal control) or a high-fat diet (HFD) either alone (HFD-control) or in combination with *G. cambogia* extract containing hydroxycitric acid (HCA, an herbal weight-loss supplement), conjugated linoleic acid (CLA, a weight-loss supplement), orlistat (a clinically available anti-obesity drug), AFW, or AFE (*n* = 6 mice per group) for 6 weeks. At the end of 6 weeks, several body weight and obesity-related parameters were examined, including: liver and adipose weight, adipocyte size, serum lipid profiles, liver expression of adenosine monophosphate-activated protein kinase (AMPK), and adipose tissue expression of uncoupling protein 2 (UCP2).

**Results:**

High-performance liquid chromatography showed that both AFE and AFW contain ferulic acid and quercetin. Oral administration of AFW and AFE to HFD-fed mice decreased body weight as well as liver and adipose tissue weight and adipocyte size. Serum lipid profiles and adiponectin levels were improved in HFD-fed mice treated with AFE but not AFW. However, both AFW and AFE significantly attenuated HFD-induced changes in serum leptin and insulin-like growth factor 1 levels, liver expression of AMPK, and adipose tissue expression of UCP2.

**Conclusions:**

The findings from this study suggest that *A. fistulosum* extracts have potential as functional food materials for weight control in obesity.

## Background

Obesity is a major risk factor for dyslipidemia, type 2 diabetes, atherosclerosis, hypertension, cardiovascular disease, and certain cancers in the developed world [1, 2]. Numerous treatments for obesity have been investigated, including the clinically available anti-obesity drug orlistat (Xenical), an intestinal lipase inhibitor [3]. However, orlistat can cause serious side effects such as insomnia, constipation, vomiting, emesis, headache, and stomachache [4]. Conjugated linoleic acid (CLA), which consists of a family of polyunsaturated fatty acids, is one of the most popular weight-loss supplements in the world [5]. However, in large doses CLA can cause fat accumulation in the liver, which can lead to metabolic syndrome and diabetes [6, 7]. Thus, there is a demand for alternative therapies, such as herbal medicines, with minimal side effects [8]. One popular weight-loss herbal supplement is *Garcinia cambogia* [9]. This edible fruit contains large quantities of hydroxycitric acid (HCA), which has fat mass loss and lipid-lowering effects [10]. *G. cambogia* protects against high-fat diet (HFD)-induced obesity by modulating adipose fatty acid synthesis and β-oxidation, but can induce hepatic fibrosis, inflammation, and oxidative stress [11].

*Allium fistulosum* (Welsh onion or bunching onion) is a species of perennial onion originated in Eastern Asia [12]. *A. fistulosum* is an important cooking ingredient in several Eastern countries, including China, Japan, and Korea [13]. In Western countries, *A. fistulosum* is used primarily as a scallion or salad onion, and is the species most commonly marketed for this purpose [14]. Its leaves have nutritional value and can be used fresh all year [15]. *A. fistulosum* also has traditional uses as an herbal medicine for the treatment for colds, influenza, abdominal pain, headache, constipation, dysentery, sores, ulcers, parasitic infestations, arthritis, and heart disease [16]. In addition, *A. fistulosum* has been shown to possess antifungal, antioxidative, antiplatelet, and antihypertensive properties [16–18]. Our previous studies showed that oral administration of *A. fistulosum*, either as a 70% ethanol extract or as a meal replacement cereal bar containing *A. fistulosum* ethanol extract, inhibited increases in body weight, adipose tissue mass, fat accumulation, and serum lipid levels in HFD-induced obese mice [19, 20]. Nutritional component analysis showed that *A. fistulosum* extract powder contains low levels of total fat and is rich in vitamins (e.g., B_2_, B_6_, niacin, and folic acid) and iron [20].

In the present preclinical study, the phytochemical contents of aqueous and ethanolic extracts of *A. fistulosum* were analyzed by high-performance liquid chromatography (HPLC)*.* The effects of these extracts on body weight and other obesity-related parameters were explored in HFD-fed mice. The anti-obesity effects of these extracts were compared with the clinical weight-loss drug, orlistat, and the weight-loss supplements, *G. cambogia* extract containing HCA and CLA.

## Methods

### Preparation of *A. fistulosum* extracts

*A. fistulosum* was purchased as a dried herb from Omniherb Co. (Yeongcheon, Korea). A voucher specimen (No. PH-79 W and PH-79E) was deposited at the herbarium of the Department of Herbal Resources Research at the Korea Institute of Oriental Medicine. The dried bulbs and roots of *A. fistulosum* (100 g) were extracted twice with 10 volumes of water or 70% ethanol using a 2-h reflux extraction at 90 °C. The extract was then concentrated under reduced pressure, filtered, lyophilized, and stored at 4 °C. Yields of the aqueous and ethanolic extracts from the starting material were 11.25% and 15.91%, respectively.

### HPLC determination of ferulic acid and quercetin

The sample was analyzed by reverse-phase HPLC using a Waters Alliance 2695 system (Waters Co., Milford, MA, USA) coupled with a 2998 photodiode array detector. An INNO C18 column (250 mm × 4.6 mm, particle size 5 μm; Young Jin Biochrom Co., Sungnam, Korea) was used as the stationary phase, and the mobile phase was composed of 0.1% (*v*/v) trifluoroacetic aqueous solution (A) and acetonitrile (B). Elution conditions were as follows. At *t* = 0 min the mobile phase consisted of 10% A/90% B and was held for 10 min. From 10 to 60 min, a gradient was applied to 60% A/40% B, which was followed by a wash with 100% B for 5 min and a 15-min equilibration period at 90% A/10% B. The separation temperature was kept constant at 40 °C throughout the analysis, with a flow rate of 1.0 mL/min and injection volume of 20 μL. Identification was based on retention time and UV spectra using comparison with commercial standards. The components were quantified based on peak areas at the maximal wavelength. Calibration curves of the standards, ranging from 3.125 to 100 μg/mL (six levels), showed good linearity, with R^2^ values exceeding 0.99 (peak areas vs. concentration). HPLC-grade reagents, acetonitrile, and water were obtained from J. T. Baker (Phillipsburg, NJ, USA). The standards of ferulic acid and quercetin (purity > 98%) were purchased from Sigma-Aldrich Co. (St. Louis, MO, USA).

### Animals and experimental diets

Male 8-week-old C57BL/6 J mice were purchased from The Jackson Laboratory (Bar Harbor, ME, USA). The mice were housed in an air-conditioned animal room with a 12-h light/12-h dark cycle at a temperature of 21 ± 2 °C and humidity of 50 ± 5%. The mice were fed a commercial diet (Ralston Purina, St. Louis, MO, USA) for 1 week, with food and water provided ad libitum.

The mice were then randomly divided into the following seven groups (*n* = 6 each) according to treatment: standard chow diet (normal control), HFD alone (HFD-control), HFD plus *G. cambogia* extract containing HCA (HFD-HCA), HFD plus conjugated linoleic acid (HFD-CLA), HFD plus orlistat (HFD-ORL), HFD plus *A. fistulosum* aqueous extract (HFD-AFW), or HFD plus *A. fistulosum* ethanolic extract (HFD-AFE). The mice receiving HCA, CLA, or ORL served as positive controls. The standard chow (Orient Bio Inc., Seongnam, Korea) provided 14% of energy as fat, 21% as protein, and 65% as carbohydrates. The HFD (Rodent Diet D12492, Research Diet, New Brunswick, NJ) provided 60% of energy as fat, 20% as protein, and 20% as carbohydrates. HCA, CLA, AFW, and AFE were dissolved in normal saline and orally administrated at a dose of 100 mg/kg/day. Orlistat (Xenical, Roche Korea Co., Seoul, Korea) was orally administered at a dose of 15.6 mg/kg, which was based on the human dosage. The normal control and HFD-control groups received vehicle (normal saline) only. Body weight and food intake were determined every week during the 6-week experimental period. The food efficiency ratio was calculated as the daily body weight gain divided by the daily food intake [21].

This study adhered to the Guide for the Care and Use of Laboratory Animals developed by the Institute of Laboratory Animal Resources of the National Research Council and was approved by the Institutional Animal Care and Use Committee of Daejeon University in Daejeon, Korea (Permit No. DJUARB2014–042).

### Serum biochemistry

At the end of the 6 weeks, the mice were anesthetized with pentobarbital (intraperitoneally, 100 mg/kg) after an overnight fasting. Blood samples were collected from the abdominal aorta into a vacuum collection tube (BD Biosciences, San Diego, CA, USA). Total cholesterol, high-density lipoprotein (HDL)-cholesterol, low-density lipoprotein (LDL)-cholesterol, triacylglycerol, free fatty acids, glucose, creatinine, aspartate transaminase (AST), and alanine transaminase (ALT) concentrations were determined using an Express Plus automatic analyzer (Chiron Diagnostics, East Walpole, MA, USA). Serum leptin, adiponectin, and insulin-like growth factor I (IGF-1) levels were determined using commercially available mouse enzyme-linked immunosorbent assay kits (Diagnostic Systems Laboratories, Inc., Webster, TX, USA and Linco Research, St Charles, MO, USA) [22].

### Histologic analysis of liver and adipose tissues

After collecting the blood, white adipose tissues (subcutaneous, epididymal, and perirenal fat) [23] and livers were removed from the mice and weighed immediately. Liver and epididymal adipose tissues were fixed in 10% neutral buffered formalin for 1 day and then embedded in paraffin. The tissues were cut (6-μm thickness) and stained with hematoxylin and eosin. Adipocyte size was determined in the stained sections using light microscopy (Olympus BX51, Olympus Optical Co., Tokyo, Japan) and an image analysis program (Image-Pro Plus 5.0, Media Cybernetics, Silver Spring, MD, USA).

### Real-time reverse-transcription polymerase chain reaction

Total RNA from liver and epididymal adipose tissue was isolated with TRI Reagent (Sigma-Aldrich) and then digested with DNase I (Life Technologies, Grand Island, NY, USA) to remove chromosomal DNA (cDNA). Total RNA (μg) was reverse transcribed into cDNA with the First Strand cDNA Synthesis Kit (Amersham Pharmacia, Piscataway, NJ, USA). Real-time reverse-transcription polymerase chain reaction (RT-PCR) was performed using the Applied Biosystems 7500 Real-Time PCR system (Applied Biosystems). The primer and probe sequences are shown in Table 1. The probes were labeled with 6-carboxyfluorescein, a fluorescent reporter dye. PCR reactions were carried out with TaqMan Universal PCR Master Mix containing DNA polymerase (Applied Biosystems) according to the manufacturer’s instructions, using the following PCR conditions: 2 min at 50 °C, 10 min at 95 °C, followed by 40 cycles of 15 s at 95 °C and 1 min at 60 °C. Relative target gene expression was determined using the comparative Ct (threshold cycle number at the cross-point between the amplification plot and threshold) method with glyceraldehyde 3-phosphate dehydrogenase as an internal control. The expression levels of mRNA were normalized to those of GAPDH and calculated using the 2^-△△Ct^ method according to the manufacturer’s instructions.

**Table 1:** The primer and probe sequences used in the real-time RT-PCR analysis

Genes	Probe and Primer	Sequence
Leptin	sense	5’-AACCCTTACTGAACTCAGATTGTTAG-3’
	antisense	5’-TAAGTCAGTTTAAATGCTTAGGG-3’
UCP-2	sense	5’-TTCAAATGAGATTGTGGGAAAAT-3’
	antisense	5’-ACCGATACAGTACAGTACAGTA-3’
Adiponectin	sense	5’-CCCAAGGGAACTTGTGCAGGTTGGATG-3’
	antisense	5’-GTTGGTATCATGGTAGAGAAGAAAGCC-3’
AMPKα2	sense	5′-GATGATGAGGTGGTGGA-3’
	antisense	5’-GCCGAGGACAAAGTGC-3’
AMPKα1	sense	5’-AAGCCGACCCAATGACATCA-3’
	antisense	5’-CTTCCTTCGTACACGCAAAT-3’
PPAR-γ	FAM	5’-TCGGAATCAGCTCTGTGGACCTCTCC-3’
GAPDH	VIC	5’-TGCATCCTGCACCACCAACTGCTTAG-3’

### Statistical analysis

Groups were compared by one-way analysis of variance followed by Duncan’s multiple range post hoc test. Results are expressed as the mean ± standard error of the mean (SEM); *p* < 0.05 was considered significant.

## Results

### HPLC analysis

**Fig. 1:**
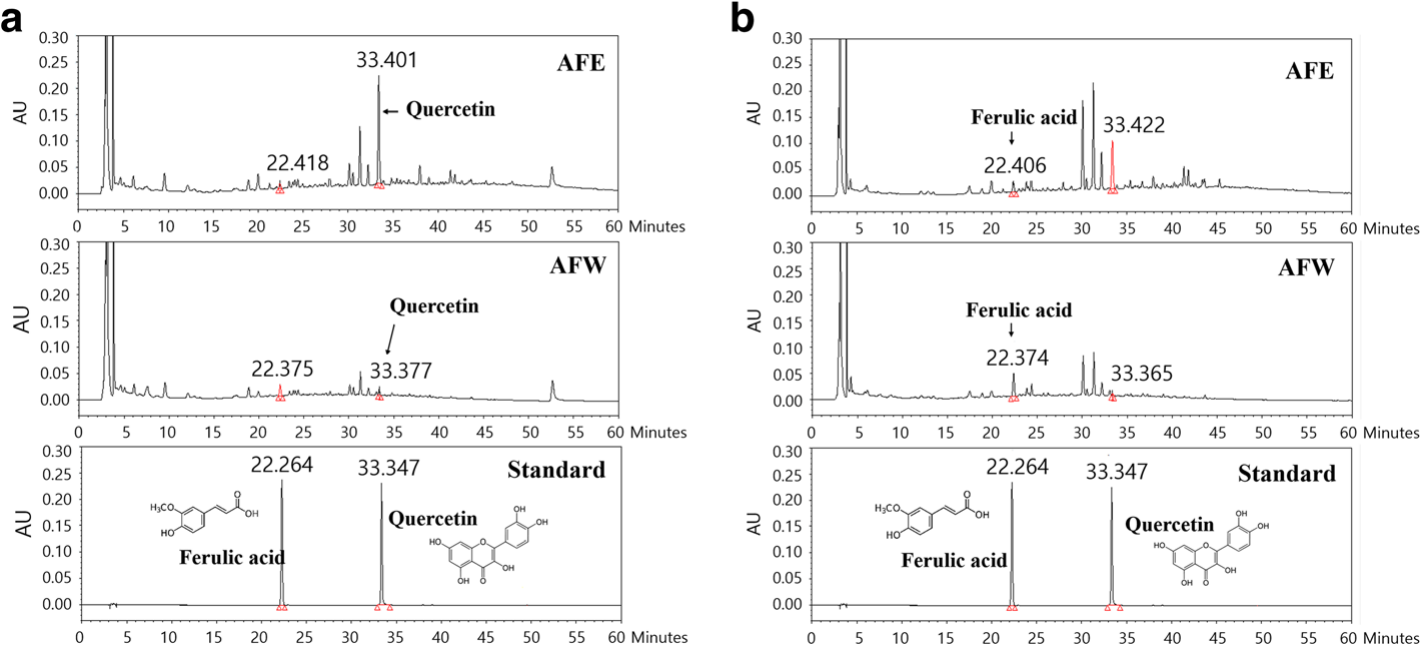
High-performance liquid chromatography/photodiode array detector chromatograms of *Allium fistulosum* aqueous extract (AFW), *A. fistulosum* ethanolic extract (AFE), and standards for quercetin and ferulic acid. Quercetin and ferulic acid were detected at (**a**) 254 nm and (**b**) 300 nm, respectively

Ferulic acid and quercetin were identified in *A. fistulosum* extracts by HPLC-photodiode array detection. As shown in Fig. 1, the retention times of ferulic acid and quercetin were approximately 22 min and 33 min, respectively. AFE contained 0.17 ± 0.02 mg/g ferulic acid and 2.22 ± 0.01 mg/g quercetin, and AFW contained 0.38 ± 0.02 mg/g ferulic acid and 0.43 ± 0.01 mg/g quercetin (Table 2).

**Table 2:** Content of components in the *A. fistulosum* extracts

	AFW (mg/g)	AFE (mg/g)
Ferulic acid	0.38 ± 0.02	0.17 ± 0.02
Quercetin	0.43 ± 0.01	2.22 ± 0.01

### Changes in body weight, food intake, and food efficiency ratio

**Fig. 2:**
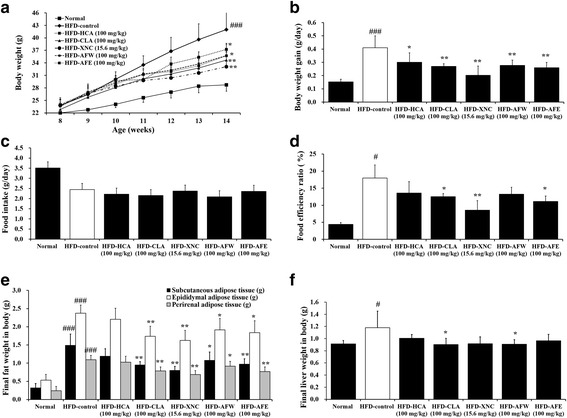
Effect of 6-week treatment with *Allium fistulosum* aqueous extract (AFW) and *A. fistulosum* ethanolic extract (AFE) in high-fat diet (HFD)-fed mice. **a** Body weight, (**b**) body weight gain, (**c**) food intake, (**d**) food efficiency ratio, (**e**) final adipose tissue weight, and (**f**) final liver weight. Normal, standard chow; HFD-control, HFD only; HFD-HCA, HFD plus 100 mg/kg *Garcinia cambogia* extract containing hydroxycitric acid; HFD-CLA, HFD plus 100 mg/kg conjugated linoleic acid; HFD-ORL, HFD plus 15.6 mg/kg Xenical (orlistat); HFD-AFW, HFD plus 100 mg/kg AFW; HFD-AFE, HFD plus 100 mg/kg AFE. Results are expressed as the mean ± standard error of the mean (SEM) (*n* = 6). # *p* < 0.05, ### *p* < 0.001 compared with the normal control group; * *p* < 0.05, ** *p* < 0.01 compared with the HFD-control group

Body weight, body weight gain, food intake, and food efficiency ratios of mice fed standard chow (normal control), HFD-control, or HFD plus HCA, CLA, ORL, AFW or AFE, for 6 weeks are shown in Fig. 1. Our results showed that the final body weight of HFD-fed mice was approximately 31.4% higher than that of normal controls (Fig. 2a). However, the final body weights of HFD-fed mice that also received HCA, CLA, or ORL (positive controls) were significantly decreased (*p* < 0.05) by approximately 11.9%, 17.4%, and 21.2%, respectively, compared with the HFD-control group. Similarly, the final body weights of HFD-fed mice that also received AFW or AFE were decreased by 14.8% and 15.0%, respectively, compared with the HFD-control group. Body weight gain also was significantly decreased in the AFW- and AFE-treated mice and in the HCA-, CLA-, and ORL-treated controls compared with the HFD-control group (Fig. 2b), although food intake did not differ significantly between the groups (Fig. 2c). The food efficiency ratio of HFD-fed mice was 4.12 times that of normal controls but was significantly decreased in HFD-fed mice that also received CLA, ORL, or AFE (38.3% lower) (Fig. 2d). The food efficiency ratio appeared to be 26.5% lower in AFW-treated mice compared with the HFD-control group, but this difference was not significant. These results suggest that AFW and AFE can inhibit HFD-induced body weight gain.

### Adipose tissue and liver weights

We found that the weights of subcutaneous, epididymal, and perirenal adipose tissues in the HFD-fed mice were 4.6, 4.5, and 4.6 times that of the normal controls, respectively. However, this HFD-induced increase in adipose tissue weight was significantly decreased by treatment with CLA, ORL, AFW, and AFE (Fig. 2e). The liver weight of HFD-fed mice was 1.3 times that of normal control mice, but this increase was significantly attenuated by treatment with CLA and AFW (Fig. 2f).

### Measurement of serum lipid and glucose levels

**Fig. 3:**
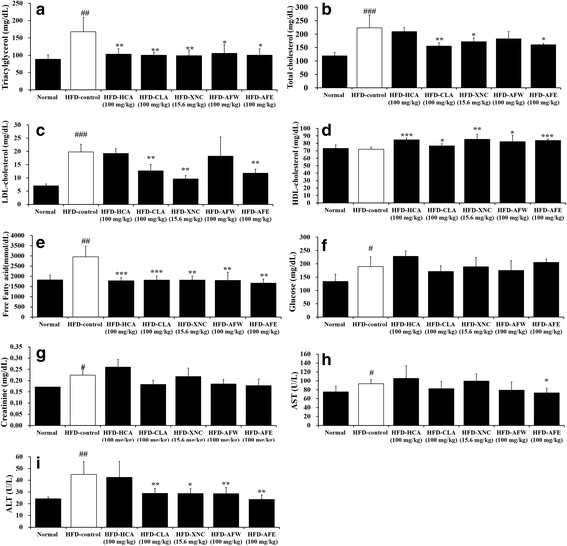
Effect of 6-week treatment with *Allium fistulosum* aqueous extract (AFW) and *A. fistulosum* ethanolic extract (AFE) on blood lipid and glucose levels and liver enzymes in high-fat diet (HFD)-induced obese mice. **a** Triacylglycerol, (**b**) total cholesterol, (**c**) LDL-cholesterol, (**d**) HDL-cholesterol, (**e**) free fatty acids, (**f**) glucose, (**g**) creatinine, (**h**) aspartate transaminase (AST), and (**i**) alanine transaminase (ALT) levels. Normal, standard chow; HFD-control, HFD only; HFD-HCA, HFD plus 100 mg/kg *Garcinia cambogia* extract containing hydroxycitric acid; HFD-CLA, HFD plus 100 mg/kg conjugated linoleic acid; HFD-ORL, HFD plus 15.6 mg/kg Xenical (orlistat); HFD-AFW, HFD plus 100 mg/kg AFW; HFD-AFE, HFD plus 100 mg/kg AFE. Results are expressed as the mean ± standard error of the mean (SEM) (*n* = 6). # *p* < 0.05, ## *p* < 0.01, ### *p* < 0.001 compared with the normal control group; * *p* < 0.05, ** *p* < 0.01, *** *p* < 0.001 compared with the HFD-control group

The effects of *A. fistulosum* extracts on serum lipid levels and glucose are shown in Fig. 3a–f. Our results show that triacylglycerol, total cholesterol, LDL-cholesterol, and free fatty acid levels were higher in HFD-fed mice compared with normal controls. Treatment with HCA, CLA, ORL, AFW, and AFE significantly attenuated the HFD-induced increase in triacylglycerol and free fatty acid concentrations (*p* < 0.05). In addition, the HFD-induced increases in total cholesterol and LDL-cholesterol levels were significantly attenuated in CLA-, ORL-, and AFE-treated mice. HDL-cholesterol level was significantly elevated in the treatment groups compared with the HFD-control group and normal controls. Glucose levels of HFD-fed mice were higher than those of the normal controls but were not significantly decreased in the mice treated with HCA, CLA, ORL, AFW, or AFE.

### Assessment of liver and kidney function

To evaluate any potential toxic effects of AFW and AFE, liver and kidney function were evaluated at the end of the 6-week experimental period (Fig. 3g–i). The level of blood creatinine, an indicator of kidney function, was increased in HFD-fed mice, but was significantly attenuated in mice treated with CLA, AFW, and AFE. AST and ALT, two indicators of liver function, also were increased in HFD-fed mice compared with normal controls. The HFD-induced increase in ALT was significantly attenuated in mice treated with CLA, ORL, AFW, and AFE; however, the HFD-induced increase in AST was significantly attenuated only in mice treated with AFE.

### Serum adipocytokine and IGF-1 concentrations

**Fig. 4:**
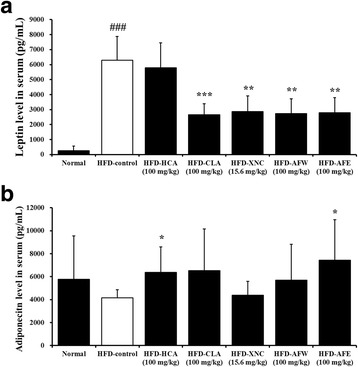
Effect of 6-week treatment with *Allium fistulosum* aqueous extract (AFW) and *A. fistulosum* ethanolic extract (AFE) on serum adipocytokine and IGF-1 concentrations in high-fat diet (HFD)-induced obese mice, as assessed by enzyme-linked immunosorbent assay. Serum levels of (**a**) leptin, (**b**) adiponectin, and (**c**) IGF-1. Results are expressed as the mean ± standard error of the mean (SEM) (*n* = 6). Normal, standard chow; HFD-control, HFD only; HFD-HCA, HFD plus 100 mg/kg *Garcinia cambogia* extract containing hydroxycitric acid; HFD-CLA, HFD plus 100 mg/kg conjugated linoleic acid; HFD-ORL, HFD plus 15.6 mg/kg Xenical (orlistat); HFD-AFW, HFD plus 100 mg/kg AFW; HFD-AFE, HFD plus 100 mg/kg AFE. ### *p* < 0.001 compared with the normal control group; * *p* < 0.05, ** *p* < 0.01, *** *p* < 0.001 compared with the HFD-control group

HFD-fed mice exhibited significantly elevated serum leptin levels compared with normal controls. This increase was attenuated by treatment with CLA, ORL, AFW, and AFE but not with HCA (Fig. 4a). Serum adiponectin levels were not significantly different in HFD-fed mice compared with controls but were increased in mice treated with HCA and AFE (Fig. 4b). Serum IGF-1 levels were significantly decreased in all treated experimental groups compared with the HFD-control group (Fig. 4c).

### Histopathological examination and gene expression in liver

**Fig. 5:**
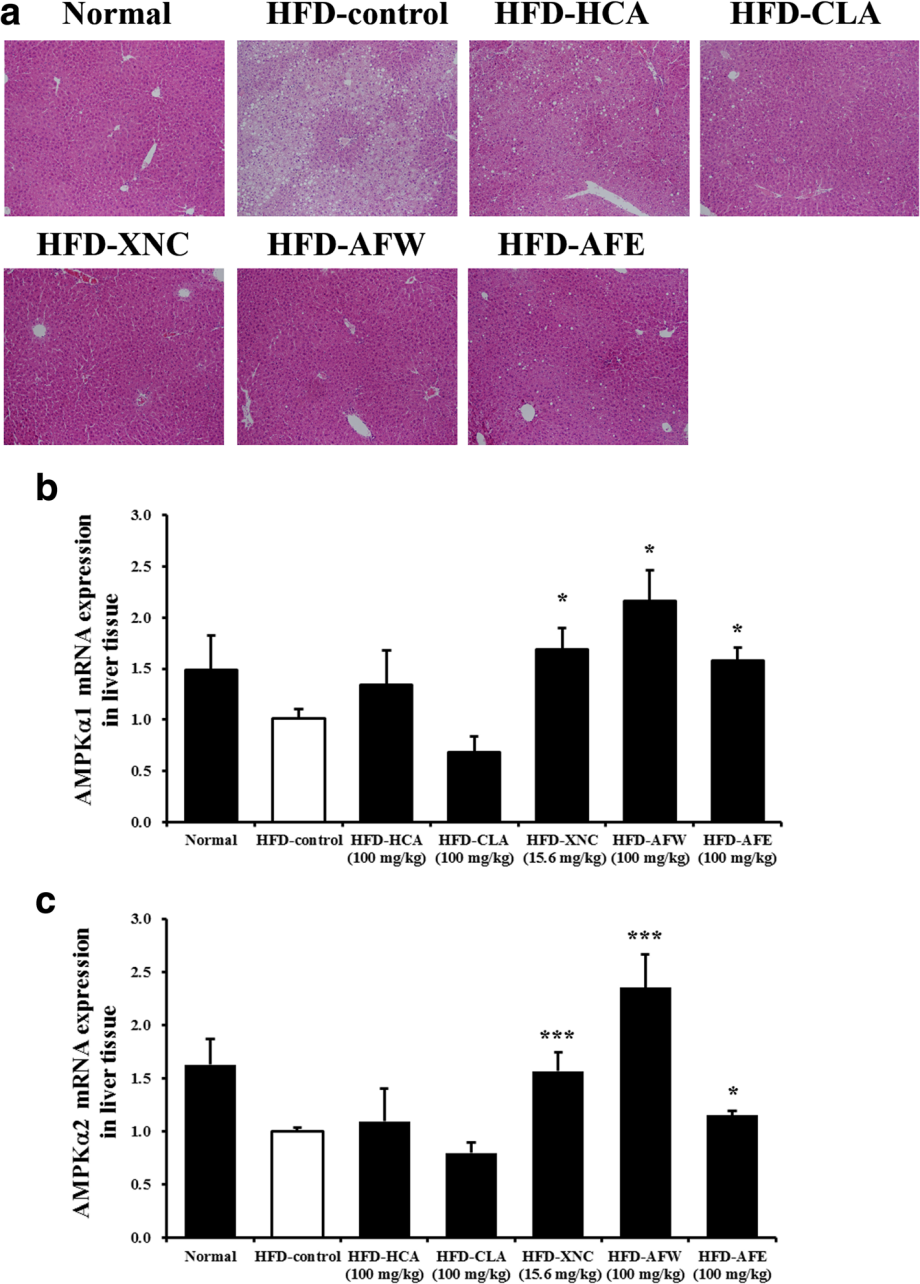
Effect of 6-week treatment with *Allium fistulosum* aqueous extract (AFW) and *A. fistulosum* ethanolic extract (AFE) on histology and gene expression in the liver tissue of high-fat diet (HFD)-induced obese mice. **a** Representative photographs of hematoxylin and eosin-stained liver sections (magnification, 400×). **b** Expression of adenosine monophosphate-activated protein kinase (AMPK) isoforms AMPKα1 and AMPKα2 mRNA in the liver, as assessed by real-time reverse-transcription polymerase chain reaction (RT-PCR). Normal, standard chow; HFD-control, HFD only; HFD-HCA, HFD plus 100 mg/kg *Garcinia cambogia* extract containing hydroxycitric acid; HFD-CLA, HFD plus 100 mg/kg conjugated linoleic acid; HFD-ORL, HFD plus 15.6 mg/kg Xenical (orlistat); HFD-AFW, HFD plus 100 mg/kg AFW; HFD-AFE, HFD plus 100 mg/kg AFE. Results are expressed as the mean ± standard error of the mean (SEM) (*n* = 6). * *p* < 0.05, *** *p* < 0.001 compared with the HFD-control group

Histologic examination of the liver showed numerous lipid droplets in the HFD-fed mice but not in the normal controls (Fig. 5a). This HFD-induced lipid accumulation was attenuated in mice that received CLA, ORL, AFW, and AFE. To identify the molecular mechanism underlying the effect of AFW and AFE on lipid accumulation in the liver, we evaluated the expression of major lipid metabolism-related genes in the liver by real-time RT-PCR. Mice treated with ORL, AFW, and AFE showed significantly increased mRNA levels of the adenosine monophosphate-activated protein kinase (AMPK) isoforms AMPKα1 and AMPKα2 compared with normal controls and HFD-control mice (Fig. 5b).

### Adipocyte size and gene expression in epididymal adipose tissue

**Fig. 6:**
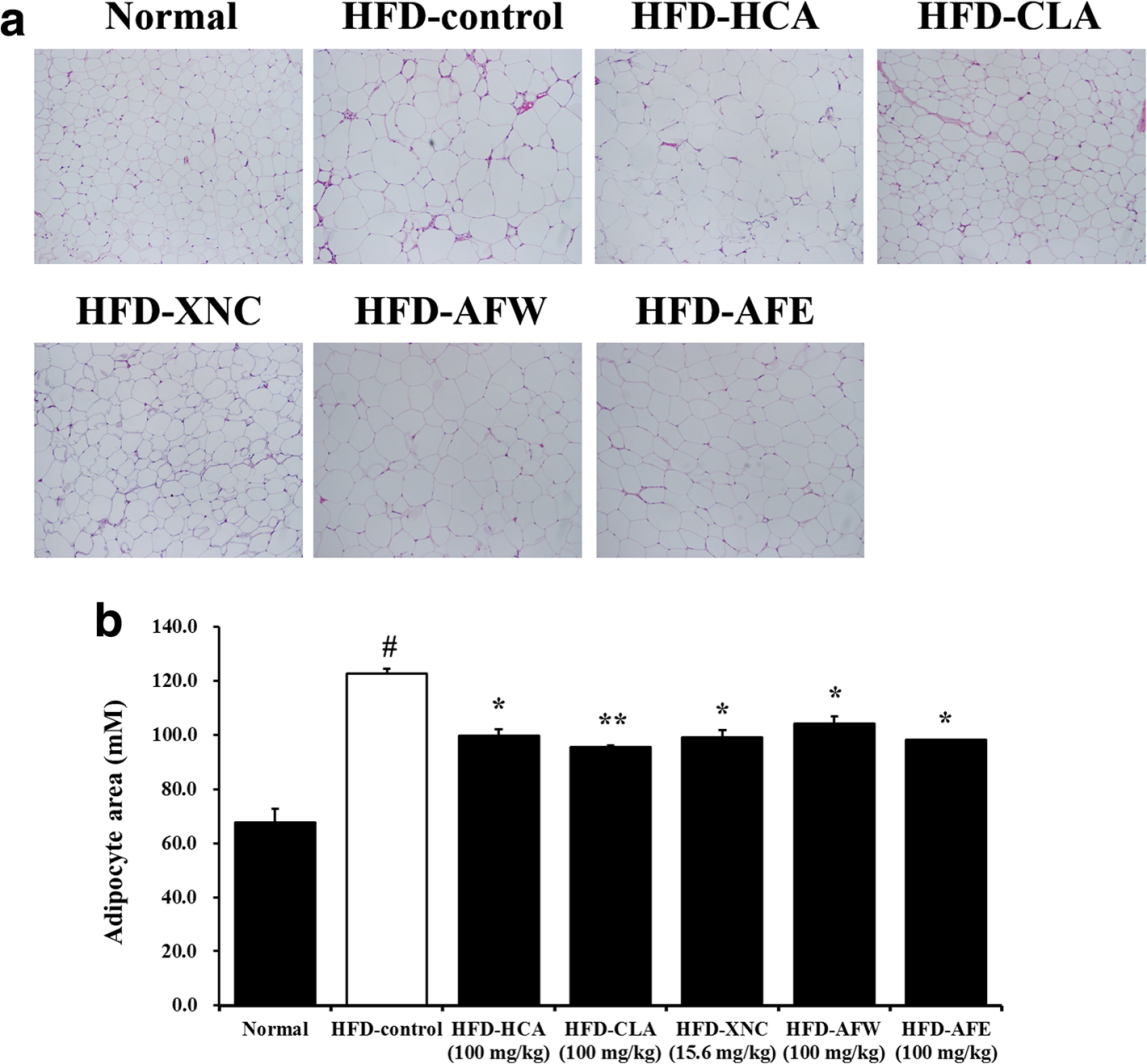
Effect of 6-week treatment with *Allium fistulosum* aqueous extract (AFW) and *A. fistulosum* ethanolic extract (AFE) on adipocyte size in epididymal adipose tissue of high-fat diet (HFD)-induced obese mice. **a** Representative photographs of adipose tissue of hematoxylin and eosin-stained tissue sections (magnification, 400×). **b** Adipocyte area of hematoxylin and eosin-stained adipose tissue sections. Normal, standard chow; HFD-control, HFD only; HFD-HCA, HFD plus 100 mg/kg *Garcinia cambogia* extract containing hydroxycitric acid; HFD-CLA, HFD plus 100 mg/kg conjugated linoleic acid; HFD-ORL, HFD plus 15.6 mg/kg Xenical (orlistat); HFD-AFW, HFD plus 100 mg/kg AFW; HFD-AFE, HFD plus 100 mg/kg AFE. Results are expressed as the mean ± standard error of the mean (SEM) (*n* = 6). # *p* < 0.05 compared with the normal control group; * *p* < 0.05, ** *p* < 0.01 compared with the HFD-control group

**Fig. 7:**
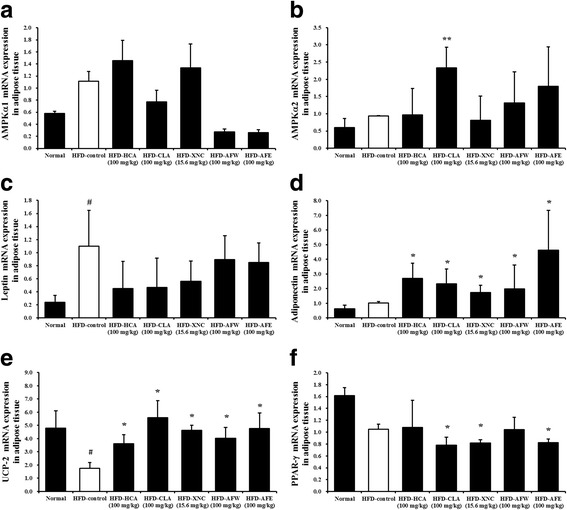
Effect of 6-week treatment with *Allium fistulosum* aqueous extract (AFW) and *A. fistulosum* ethanolic extract (AFE) on gene expression in epididymal adipose tissue of high-fat diet (HFD)-induced obese mice. Expression levels of (**a**) adenosine monophosphate-activated protein kinase (AMPK) isoforms AMPKα1, (**b**) AMPKα2, (**c**) leptin, (**d**) adiponectin, (**e**) uncoupling protein 2 (UCP2), and (**f**) peroxisome proliferator-activated receptor gamma (PPAR-γ), as assessed by real-time reverse-transcription polymerase chain reaction (RT-PCR). Normal, standard chow; HFD-control, HFD only; HFD-HCA, HFD plus 100 mg/kg *Garcinia cambogia* extract containing hydroxycitric acid; HFD-CLA, HFD plus 100 mg/kg conjugated linoleic acid; HFD-ORL, HFD plus 15.6 mg/kg Xenical (orlistat); HFD-AFW, HFD plus 100 mg/kg AFW; HFD-AFE, HFD plus 100 mg/kg AFE. Results are expressed as the mean ± standard error of the mean (SEM) (*n* = 6). # *p* < 0.05 compared with the normal control group; * *p* < 0.05, ** *p* < 0.01 compared with the HFD-control group

Examination of epididymal adipose tissue sections showed significantly increased adipocyte size in the HFD-control mice compared with normal controls (Fig. 6a). The increase in adipocyte area was attenuated by HCA, CLA, ORL, AFW, and AFE (Fig. 6b). Results of real-time RT-PCR showed that mRNA levels of AMPKα1 and AMPKα2 in epididymal adipose tissue were not significantly higher in HFD-control mice compared with normal controls (Fig. 7a and b). Leptin mRNA levels were significantly higher in HFD-control mice compared with normal controls. This increase was not significantly attenuated by treatment with HCA, CLA, ORL, AFW, and AFE (Fig. 7c). In contrast, adiponectin mRNA levels were significantly higher in all treatment groups compared with normal controls and HFD-fed controls (Fig. 7d). Uncoupling protein 2 (UCP2) mRNA levels were significantly lower in HFD-fed mice compared with normal controls, but the HFD-induced decrease was significantly attenuated by all treatments (Fig. 7e). Peroxisome proliferator-activated receptor gamma (PPAR-γ) mRNA levels were decreased in mice receiving CLA, ORL, and AFE compared with normal controls and mice receiving the HFD only (Fig. 7f).

## Discussion

In the present study, oral administration of AFW and AFE attenuated the HFD-induced increase in serum levels of triacylglycerol, free fatty acids, and leptin; lipid accumulation in the liver; liver and adipose tissue weight; adipocyte size; and body weight in mice. In addition, AFE attenuated the HFD-induced increase in total cholesterol and LDL-cholesterol. We found that serum HDL-cholesterol and adiponectin levels were higher in HFD-fed mice treated with AFW and AFE than in normal controls and mice receiving the HFD only.

The insulin/IGF-1 axis plays an important role in the association between obesity and risk of breast cancer [24]. Some studies have demonstrated that obese children exhibit the highest levels of serum IGF-1 and also exhibit a positive relationship between IGF-1 and adiposity, which perhaps contributes to their increased risk of obesity-related cancers [25]. In this study, the HFD-induced increase in serum IGF-1 levels was significantly attenuated in mice treated with AFW and AFE. Expression of AMPK, which is a cellular energy sensor involved in energy homeostasis, was evaluated in adipose tissue and liver. AMPK triggers catabolic pathways, such as glucose transport and fatty acid β-oxidation, and inhibits anabolic pathways such as fatty acid, cholesterol, and protein synthesis [26]. Because of its critical role in metabolism, AMPK is a target for drugs used to treat cancer, diabetes, and metabolic syndrome [27]. Our results show that treating HFD-fed mice with AFW or AFE increased mRNA levels of AMPKα in the liver, suggesting that AFW and AFE may lower lipid accumulation and serum lipid levels through the regulation of lipid metabolism in the liver. In adipose tissue, AFW and AFE increased UCP2 and adiponectin expression. In addition, AFE decreased the expression of PPAR-γ, which is a ligand-activated transcription factor that plays an important role in adipocyte differentiation and lipid storage [28] and regulates the expression of adiponectin through a PPAR-responsive element in its promoter [29]. UCP2, a mitochondria inner membrane transporter, promotes fatty acid oxidation in adipose tissue and lowers body weight [30].

Adiponectin is a hormone secreted by adipose tissue that modulates glucose and lipid metabolism by stimulating fatty acid oxidation in almost all its major target tissues, including skeletal muscle, liver, and adipocytes [31]. The beneficial effects of adiponectin in these target tissues are dramatically attenuated in UCP2-deficient mice and by expression of dominant negative AMPK [32]. In patients with obesity and diabetes, plasma adiponectin levels are inversely correlated with body fat content, suggesting that adiponectin is an important factor for the treatment of obesity [33, 34]. In the present study, it was found that serum adiponectin levels and adiponectin mRNA levels in adipose tissue were higher in AFE-treated mice compared with mice receiving the HFD only. Conversely, the HFD-induced increase in serum leptin levels was significantly attenuated in mice treated with AFW and AFE, although leptin mRNA levels in adipose tissue were not significantly decreased in the treatment groups. Blood levels of leptin are positively associated with adiposity and increased body weight in humans and rodents [35]. These results suggest that the regulation of adiponectin and lipid metabolism-related genes expression by AFW and AFE may be responsible for the decrease in serum lipid levels and the attenuation of adiposity in HFD mice.

HPLC analysis identified ferulic acid and quercetin in AFE and AFW. Ethanol and water are the most common solvent to extract the bioactive compounds of herbal medicine for HPLC analysis and traditional decoction, respectively. Our HPLC analysis identified ferulic acid and quercetin in AFE and AFW. The ferulic acid concentration in AFW was 2 times that of AFE and the quercetin concentration in AFE was 5.2 times that of AFW. The HPLC analysis was performed using commercial standards and compound libraries. Other compounds containing vanillic acid, tyrosol, 4-hydroxybenzaldehyde, and koumine were detected in the small peaks, but the content of these compounds in AFE and AFW was extremely low. Quercetin and ferulic acid, which are naturally occurring polyphenolic flavonoid compounds in fruits and vegetables, have been reported to suppress body weight, fat accumulation and hyperlipidemia in obese mice by enhancing antioxidant activities [36, 37]. The anti-obesity effect of quercetin is mediated by the AMPK signaling pathway in 3 T3-L1 adipocytes [38]. Structurally, hydroxyl groups of these flavonoids participate in radical stabilization via electron delocalization, thus contributing to the strong antioxidant and anti-obesity activities [39–41]. These phenolic compounds therefore may be partly responsible for the anti-obesity effects of AFW and AFE. In this study, especially, the quercetin concentration in AFE was high. Among the flavonoids, quercetin is an effective protector against lipid oxidation due to the presence and location of five hydroxyl groups on the aromatic ring [42]. Additionally, AFE exerted the stronger anti-obesity effects than AFW by enhancing adiponectin levels and reducing serum cholesterol levels in obese mice. Thus, this difference in anti-obesity action might be attributed to their chemical difference of quercetin.

However, it cannot be excluded that other components of these extracts may contribute to their anti-obesity activities. It has been reported that *A. fistulosum* contains *p*-coumaric acid, isoquercitrin, quercitrin, stigmasterol, β-sitosterol, campesterol, and allicin [15, 43]*.* Thus, further study is needed to determine the anti-obesity components of *A. fistulosum*, including the development of optimized methods of chemical analysis to identify the bioactive compounds in the AFE and AFW extracts.

## Conclusions

This study showed that the oral administration of AFW and AFE significantly reduced body weight, adipose tissue and liver weights, and fat accumulation in a mouse model of obesity. In addition, AFW and AFE improved the serum levels of triacylglycerol, total cholesterol, LDL-cholesterol, free fatty acids, and HDL-cholesterol. These findings indicate that *A. fistulosum* may be useful as a functional food material or therapeutic agent for the treatment of obesity and obesity-associated metabolic syndrome.

## Abbreviations

AFE: *A. fistulosum* ethanolic extract
AFW: *A. fistulosum* aqueous extract
ALT: Alanine aminotransferase
AMPK: AMP-activated protein kinase
AST: Aspartate aminotransferase
CLA: Conjugated linoleic acid
HCA: *Garcinia cambogia* extract containing hydroxycitric acid
HDL: High-density lipoprotein
HFD: High-fat diet
HPLC: High-performance liquid chromatography
IGF: Insulin-like growth factor
ORL: Orlistat
PPAR-γ: Peroxisome proliferator-activated receptor-γ
RT-PCR: Reverse-transcription polymerase chain reaction
SEM: Standard error of the mean
UCP2: Uncoupling protein 2
